# The new paradigm of economic complexity^✰^

**DOI:** 10.1016/j.respol.2021.104450

**Published:** 2022-04

**Authors:** Pierre-Alexandre Balland, Tom Broekel, Dario Diodato, Elisa Giuliani, Ricardo Hausmann, Neave O'Clery, David Rigby

**Affiliations:** a Department of Economic Geography, Utrecht University, The Netherlands; b Center for Collective Learning, Artificial and Natural Intelligence Toulouse Institute, France; c University of Stavanger Business School, Stavanger, Norway; d European Commission, Joint Research Centre (JRC), Seville, Spain; e Responsible Management Research Center, University of Pisa, Italy; f Growth Lab, John F. Kennedy School of Government, Harvard University, USA; g Centre for Advanced Spatial Analysis, University College London, UK; h Departments of Geography and Statistics, UCLA, USA

**Keywords:** Economic complexity, Complex systems, Innovation, Policy, Geography

## Abstract

Economic complexity offers a potentially powerful paradigm to understand key societal issues and challenges of our time. The underlying idea is that growth, development, technological change, income inequality, spatial disparities, and resilience are the visible outcomes of hidden systemic interactions. The study of economic complexity seeks to understand the structure of these interactions and how they shape various socioeconomic processes. This emerging field relies heavily on big data and machine learning techniques. This brief introduction to economic complexity has three aims. The first is to summarize key theoretical foundations and principles of economic complexity. The second is to briefly review the tools and metrics developed in the economic complexity literature that exploit information encoded in the structure of the economy to find new empirical patterns. The final aim is to highlight the insights from economic complexity to improve prediction and political decision-making. Institutions including the World Bank, the European Commission, the World Economic Forum, the OECD, and a range of national and regional organizations have begun to embrace the principles of economic complexity and its analytical framework. We discuss policy implications of this field, in particular the usefulness of building recommendation systems for major public investment decisions in a complex world.

## Highlights

• Economic complexity offers a potentially powerful paradigm to understand key societal issues and challenges of our time.
• This brief introduction to economic complexity summarizes key theoretical foundations and principles of economic complexity.
• It also reviews the tools and metrics developed in the economic complexity literature that exploit information encoded in the structure of the economy to find new empirical patterns.
• It finally aims is to highlight the insights from economic complexity to improve political decision-making.

## 1 Introduction

The central point of economic complexity is that some of the biggest societal issues of our time only start to make sense if we look at the systemic interactions that produce them. As an example of a systematic interaction, let us think about Google. Google's monopoly over internet search goes beyond having the smartest engineers, the largest R&D investments, or the best AI. It is the outcome of a self-reinforcing feedback loop in which slightly better predictions attract more users, which in turn provides more data, leading to better predictions. Iterate enough times and you end up with a snowball effect that promotes exponential adoption and control over the internet search market. The same idea applies to individual returns – returns that are increasingly decoupled from talent and effort in a complex society – and instead based on the leverage of complex economic interactions originating from the division of labor, capital flows, media presence, and task automation.

The analysis of economic complexity offers a broad framework that can be applied to many societal challenges. In this introduction to the Special Issue we focus on matters relating to technology and innovation. Our discussion begins by exploring how humans managed to divide the process of knowledge production and organize complex interdependencies that, in turn, create extraordinary technologies.

### 1.1 From the division of human knowledge to extraordinary technologies

The far-reaching extent of human technology never ceases to surprise us and capture our imagination. We have harnessed the quantum mechanical quirks of semiconductors to make microprocessors. We use the general theory of relativity to calculate GPS positions. We use micro-biology to leaven bread, ferment beer and wine and produce vaccines. We use material science to create touchable screens. We can only be mesmerized by how quickly intelligent machines have learned to drive cars, read lung scans, or predict what we will want to listen to, watch, or buy better than any human. But where does all of this knowledge reside? What determines how and where it is put to use? How does it grow?

The framework of economic complexity is intrinsically entangled with these questions. A way of illustrating how the research universe of economic complexity arises from a central understanding of technology is to posit that productive knowledge takes three forms: embodied knowledge in tools and materials or artifacts, codified knowledge in books, formulas, algorithms and how-to-do manuals, and tacit knowledge or know-how in brains. Tools exist in 3-dimensional space and can be transported. Codes exist in some symbolic space. Although they can be represented as ink on paper or pixels on a screen, their meaning is not in the material but in the symbols they represent. As such, they can be shared using the many communication technologies we have available. Know-how resides in brains and only in brains.^1^ It moves with enormous difficulty from brain to brain because it is unconscious and does not involve understanding. Examples are our ability to walk, to ride a bike, to use language or to infer a person's intentions from their facial expressions. We know how to do these tasks, but we do not know what it is that we do when we do them and hence, we do not know how to teach others to do them. This is part of what Kahneman and Tversky called System 1 (see Kahneman, 2011). This know-how resides in the wiring of our neurons, a result of a long process of repetition, imitation and feedback. While, during the Renaissance, it was conceivable, if not common, that gifted polymaths excelled in multiple disciplines, the world's knowledge has since grown too much for a single person to even master one field. Today, only the division of knowledge across many individuals allows us to overcome individual human limits and makes the technological progress of modern societies possible (Jones, 2009).

Several implications emerge from this. First, in the short run, tools, codes and know-how are very strong complements: to drive from home to work we need the car (the tool), we need the layout of the transportation network and its many rules (the codes) and we need to know how to drive the car and how to identify where we are in space (the know-how). We need all three to complete the task. Over time, things may change. Software applications may make driving less reliant on know-how by moving information and interpretation to a tool and self-driving cars may do away with the driver altogether. But at any moment in time tools, codes and know-how are strongly complementary. This implies that the implementation of a particular technology at a given point in space (i.e., its geographic diffusion) is bound to be limited not by the absence of tools or codes, which are relatively easy to move, but by the absence of the requisite know-how.

Second, given the limitations of how much know-how fits in a person, the growth of know-how at the societal level occurs at the extensive margin^2^ through the division of tacit knowledge between individuals: *the whole knows more because individuals know different,* which is to say that the growth of know-how happens thanks to specialization*.* Specialization and diversification are, in fact, two aspects of the same phenomenon seen from two different scales. If individuals specialize, firms, cities, and countries diversify. Yet, many scholars and policymakers have wrongly equated the benefits of individual specialization with those of specialization at higher scales (Hausmann, 2013). This is an example of the fallacy of composition. As a matter of fact, the opposite is true: societies with very specialized individuals have access to a greater variety of knowledge and are, therefore, more diversified*. “It is the great multiplication of the productions of all the different arts, in consequence of the division of labor, which occasions, in a well-governed society, that universal opulence which extends itself to the lowest ranks of the people”* writes Adam Smith, making this point for the first time in 1776 (Smith, 1776, pp.18–19). Numerous empirical studies confirm that developed nations are, in fact, more diversified, supplying greater varieties of products and services (Imbs and Wacziarg, 2003; Bustos et al., 2012; Tacchella et al., 2013; Bettencourt et al., 2014; Petralia et al., 2017).

Third, there are dynamic effects of specialization: not only does it enable societies to accumulate more collective knowledge, but it also allows them to create new knowledge. As invention has been described by many authors as the discovery of new useful combinations of existing ideas (Weitzman, 1998; Fleming and Sorenson, 2001), a society of highly specialized individuals, one that has a greater variety of expertise, is more likely to be able to combine old ideas into new technologies. This combinatorial aspect of the division of knowledge has serious economic consequences. Hausmann and Hidalgo (2011) argue that, as the number of possibilities grows exponentially with the variety of elements to combine, countries with few (many) of those elements will have weak (strong) incentives to accumulate more elements as they may produce few (many) new combinations. This may cause a poverty trap^3^ that is responsible, at least in part, for the Great Divergence of incomes observed over the past two centuries (Pritchett, 1997).

This directly leads to *the* core principle of the literature on economic complexity: not only are developed nations more diversified, they are also more complex. That is, developed nations are capable of supplying products or services that require a greater variety (hence a greater amount) of knowledge. Goods and services differ in the amount of knowledge they require and hence in the variety and types of tools, codes and know-how that must be available for their production. For example, internet retailing presumes the local availability of internet services, electronic payments systems and a distribution network. It also presumes a team of people who know about IT, design, marketing, finance, accounting, procurement, contracts and after-service care. This means that, for a place to be able to make a particular good or service, it must be able to assemble the required knowledge components. The most complex products or technologies are highly leveraged items that everybody wants but very few know how to produce.

### 1.2 The contribution of economic complexity to economics and related disciplines

The new approaches to economic complexity discussed below are clearly related to complexity in other areas of science and those new approaches can benefit from and contribute to complexity developments in those other fields. At one level, the brain (society) is composed of very similar neurons (humans) but its capabilities emerge from specialization and interconnections, making the idea of a social brain more than just a metaphor. Ecological systems involve specialized species that interact through trophic, mutualistic and other connections. Locations differ in their diversity and species differ in their ubiquity. Moreover, more diverse ecosystems tend to host less ubiquitous species, just as in economic systems. No wonder many of the methods developed in ecology have been close to the ones that have proven useful in economic complexity.^4^

The notion of economic complexity adds to the toolbox of economics in at least two ways. First, it expands the methods available to reduce the dimensionality of a problem in order to study it.^5^ A common approach in economics has been to aggregate data: for example, national accounts use firm, household, government and customs data to calculate aggregates such as gross domestic product, investment, consumption, exports and imports. In this process, information is collapsed by adding up different entries. Economic complexity uses methods of spectral analysis and network theory to reduce the dimensionality of the data in ways that preserve more information than mere aggregates. Measures of economic complexity, such as the Economic Complexity Index (ECI) (Hidalgo and Hausmann, 2009), Fitness (Tacchella et al., 2012) or production ability introduced in this Special Issue by Bustos and Yildirim (2021) are examples of spectral methods. The product/industry/technology/occupation spaces that have been developed in the relatedness and complexity literature are examples of methods that use information on locations and activities to estimate new measures of proximity between activities and locations that are then studied as weighted networks.

Secondly, economics has had difficulty in studying technology. It has tended to measure it through its consequences: as a shift parameter in aggregate production functions such as measures of total factor productivity (see the survey in Hulten, 2001). But it does not provide a connection from its consequences to its causes, which may be contained in information erased through data aggregation. Rich countries are not just like poor countries that get more output out of their capital and labor inputs: they produce radically different things using radically different methods of production. What they produce is in the data before aggregation. Economic complexity methods allow us to reduce data dimensionality, while still capturing information about what countries produce —which has been shown to be important for our understanding of productivity, income and growth.

Adam Smith's idea that productivity is related to the division of labor has been captured in endogenous growth theories, such as Grossman and Helpman (1991) and Aghion and Howitt (1992) .^6^ These papers use a Dixit-Stiglitz production function which treats products as equally substitutable for one other. This is clearly an assumption that simplifies the algebra but is very far from reality. In Ricardian models, following Eaton and Kortum (2002), a country's comparative advantage in a particular product comes from random draws from an extreme value distribution, with no correlation across draws. This means that the probability of having a comparative advantage in aircraft manufacturing is unrelated to whether the country is currently producing coffee or cars. Economic complexity methods can make a contribution by making it feasible to introduce relevant heterogeneity between industries and products (in terms of their complexity, connectedness and relatedness) in a way that is simple, tractable and empirically implementable. For example, Mealy and Teytelboym (2021) in this Special Issue use economic complexity measures to map out the growth possibilities of countries in the green economy. Likewise, Atkin et al. (2021) embed economic complexity measures in a Ricardian model of trade to explore the impact of trade on growth through its impact on capability accumulation. Many other applications of the concept of economic complexity are explored in the following discussion.

### 1.3 Outline

In this Introduction to the Special Issue on Economic Complexity, our goals are to highlight the recent rebirth of interest in this topic stemming from the important work of Hidalgo and Hausmann (2009). Throughout the discussion we hope to convey the general importance of the concept and its broad applicability. The core arguments are divided into six parts of Section 2. These arguments outline a general framework for thinking about economic complexity and they move on to target issues of measurement, the links between complexity and economic performance, the spatial concentration and geographical scales of complexity, the concept of relatedness and the division of knowledge. Section 3 offers a brief conclusion highlighting some of the policy implications that derive from our understanding of economic complexity.

## 2 Themes in economic complexity

### 2.1 A general framework for studying economic complexity

To parsimoniously introduce the disparate themes, issues, and open questions that are at the core of economic complexity analysis, we describe the core principles of this literature through a compact mathematical notation. Key metrics in the economic complexity literature have been based on the idea that it is possible to extract useful information from data on the spatial distribution of economic activities. This is achieved by organizing information on what countries (*c*) produce particular products (*p*) into a matrix with dimensions *c* × *p.* Typically, this matrix is denoted as **M***_cp_* and elements in the matrix assume a value of 1 if a country produces a product above a given threshold (and zero otherwise).

The accumulation of technological know-how within a country is expected to lead to the diversification of production and an increase in the capacity of the economic agents within the country to produce complex products. If we assume that economic agents within countries are endowed with certain capabilities and that products require specific capabilities for their production, we can describe the relation between know-how and output with the following formula:

$$M_{cp}=C_{ca}⊙P_{pa}$$

Capabilities are captured by an endowment matrix **C** with dimensions *c* × *a,* where *a* stands for ability (some form of know-how). Technology, the process by which we use our abilities to make things, is captured by the matrix **P**. Together these two matrices determine who makes what (the location vs. product matrix, **M**_*cp*_). The operator ⊙ indicates the relationship between the local availability of capabilities and actualization of production, given technological requirements. In early work, the operator ⊙ was assumed to be Leontief, meaning that a country or location would make a product if the product's vector of capability requirements in the **P***_pa_* was a subset of the country's capability endowment in the **C***_ca_*. More recent papers, such as Gomez-Lievano et al. (2016) assume this relationship to be stochastic, where the completeness of the capability endowment affects the probability that a product will be made.

A core presumption that kickstarted the economic complexity literature is that, since we have partial knowledge (at best) of the process implied in **C***_ca_* ⊙ **P***_pa_*, we can derive the amount of technological know-how present in a country directly from the **M***_cp_* matrix. Economic complexity indices are, in fact, attempts to use **M***_cp_* to derive general measures of complexity for countries (informative of **C***_ca_*) and products (informative of **P***_pa_*). The key to this inference is that we can extract useful information from the diversity of countries’ production, as well as from the ubiquity of products.

Consider the following two stochastic matrices: **A** and **B**

$$A_{cp}=D_{cc}M_{cp}$$

$$B_{cp}=M_{cp}U_{pp}$$

where **D***_cc_* and **U***_pp_* are diagonal matrices. The entries of **D***_cc_* are the inverse of the row sums of the **M***_cp_*, i.e., the diversity of the country. The entries of **U***_pp_* are the inverse of the column sums of the **M***_cp_*, i.e., the ubiquity of the product. The original Economic Complexity Index (ECI, Hidalgo and Haumann, 2009) is the eigenvector associated with the second largest eigenvalue of the matrix **A***_cp_* **B***_cp_*^T^ and the Product Complexity Index (PCI) is the eigenvector associated with the second largest eigenvalue of the matrix **A**_*cp*_^T^ **B**_*cp*_.

A large literature has developed around the alternative vectors that can be extracted from the **M***_cp_* matrix by incorporating different weighting schemes. In Section 2.2, we review these efforts, along with the rich discussion around the interpretations of the different indices. In Section 2.3, we discuss how these vectors have been successfully applied in different empirical contexts. Up to this point, we have assumed that the dimensions of the **M***_cp_* matrix are countries and products, as used to compute the first ECI. However, subsequent literature has explored a variety of alternative dimensions both for locations (e.g., states, cities) and activities (e.g. industries, technology classes, scientific fields). In Section 2.4 we focus specifically on the geographical dimension of complexity: what is the relevant scale at which capabilities come together to synthesize a product or a service? The literature in economic geography, which suggests knowledge is primarily generated and coordinated in cities and regions, has inspired scholars to explore economic complexity dynamics at the sub-national scale.

Another important stream in the economic complexity literature revolves around the concept of the product space (Hidalgo et al., 2007), which can be thought of as a measure of similarity or relatedness of two products in the **P***_pa_*. This can be inferred from the **M***_cp_* matrix because if two products have similar rows in the **P***_pa_*, countries that are able to do one of them should also be able to do the other. Hence the probability that two products are co-exported by the same countries is informative of their similarity. Symmetrically, one can think of a country space, where countries are related if they have a similar endowment of capabilities in the **C***_ca_* and this would be reflected in the **M***_cp_*. While several alternative metrics are possible, we can use our compact framework to define the following product space (**PS**) and country space (**CS**) matrices:

$$PS_{\mathrm{pp}}={A_{\mathrm{cp}}}^{T}B_{\mathrm{cp}}$$

$$CS_{\mathrm{cc}}=A_{\mathrm{cp}}{B_{\mathrm{cp}}}^{T}$$

The product space captures the proximity between the input vectors in the **P***_pa_* and the country space should capture the proximity of countries in the **C**_ca_.^7^ One of the most important empirical applications of the product space is in growth predictions. The distance between a location and an industry in capability space can be measured as **M***_cp_* **PS**_*pp*_ and it is a robust predictor of **M***_cp_* in a subsequent period, both at the extensive margin (ie the appearance of industries not present before) and at the intensive margin (i.e., when **M***_cp_* is a continuous measure of presence, such as output in dollars, rather than a dummy variable). In Section 2.5 we review the relatedness and product space literature.

The bulk of economic complexity analysis uses some variant of the **M***_cp_* matrix. But this means that the analysis is carried out at the “phenotypic” level (i.e., the level of countries and products), without knowledge of the “genotypic” level, which presumes knowledge of **C***_ca_* and **P***_pa_*, or even of the operator ⊙. Yet, the conception of technology based on the division of labor, which we presented in Section 1.1, is largely based on these “genotypic” elements. What are these skills, capabilities, know-how that we aggregate in index *a*? How does technological progress – for instance the embedding of know-how into an automatic tool – change skill requirements embedded in matrix **P***_pa_*? How do workers with different capabilities interact and coordinate with each other to make the final product – that is, what is the operator ⊙?

In Section 2.6, we discuss how the division of labor, once only used in economic complexity as a powerful idea to justify “phenotypic” analysis, is increasingly becoming fertile research ground in the literature, giving rise to several new research branches at the “genotypic” level.

### 2.2 Complexity measurement

As introduced above, Hidalgo and Hausmann (2009) devised a method to capture the complexity of individual products and countries by looking at the global pattern of exports. The general idea proposed is that complex products are rare (i.e., have low ubiquity), and found only in places that produce many other products (i.e., are highly diversified). The PCI is a metric derived from a bipartite network of products and countries (encoded in **M***_cp_*), and is calculated using the ‘Method of Reflections’. This functions by recursively computing the average diversity of countries that make a specific product, and the average ubiquity of the other products that these countries produce. Hence, a product is considered complex if it is produced by a few highly diversified countries that produce products that are themselves rare and made by highly diversified countries. A country is complex, according to the ECI, if it produces many products (i.e., is highly diversified), especially those that are relatively rare (having low ubiquity). As this calculation is iterated, it converges to the second eigenvector of the **A**_*cp*_ **B**_*cp*_^T^ matrix. The final value of a country's ECI is the average PCI of the products that it exports, and the PCI of an individual product is the average ECI of the countries that export that product. Hidalgo and Hausmann (2009) and Hausmann et al. (2014) show that the ECI values for countries are highly correlated with GDP per capita, especially after controlling for natural resource wealth. More importantly, deviations in scatterplots of country ECI and GDP per capita values at any time predict future growth, indicating that countries tend to converge to a level of income that is determined by their ECI.

While widely adopted within both the academic and policy literature (see below for a discussion on policy), the somewhat complicated formulation of the ECI and PCI measures has led to difficulties in interpretation. Addressing this issue, Mealy et al. (2019) note that the ECI is in fact the Fiedler eigenvector and is hence equivalent to a two-dimensional embedding of the matrix **A**_*cp*_^T^ **B**_*cp*_ or, equivalently, a network with edge weights corresponding to product co-presences. This vector is commonly deployed as a spectral clustering algorithm that partitions a network into two parts: the entries of the eigenvector can be seen as a ‘distance’ to the split. From this perspective, the ECI and PCI can be seen as measures of country and product clustering.

There have been a variety of adaptations of the ECI approach. In particular, Tacchella et al. (2012) and Cristelli et al. (2013) critique the ECI method based on how it estimates the complexity of a product. Their key argument is that the complexity of a product cannot be defined as the average complexity of the countries producing it (as is the case for PCI) since high complexity countries make nearly all products while low complexity countries only make low complexity products. The authors propose a refinement of the ECI method, defining the ‘Fitness’ of a country (and ‘Quality’ of a product) based on an iterative scheme that weighs more heavily low Fitness exporters when estimating the Quality of a product. Some methodological issues with the stability or convergence of this algorithm have been pointed out (Morrison et al., 2017) and improved (Servedio et al., 2018). More recently, Sciarra et al. (2020) united these methods (ECI and Fitness) under a common mathematical framework, and proposed a new metric, the GENEPY index, which combines information on both ECI and Fitness. The authors argue that one advantage of this index is that it can be easily interpreted and computed as a standard node centrality measure derived from a product similarity network.

In general, one can see ECI, Fitness and GENEPY as members of a large family of metrics for complexity derived from spatial patterns of production. Brummitt et al. (2020) took an agnostic approach as to whether a single measure can adequately describe economic performance, and developed a machine learning algorithm to identify the main statistical patterns in the evolution of country export baskets. Their algorithm revealed a quantity that captured more than 50% of the variance in export baskets over fifty years of data, which the authors show is a “complexity-weighted” measure of diversity.

While these metrics are typically derived from cross-sectional spatial patterns of production, several recent models aim to construct a complexity metric based on a probability model for temporal capability accumulation. For example, O'Clery et al. (2021) developed a method to uncover ’product ecosystems’, the set of products that need to be present for a new product to appear in the export basket of a country. The authors develop a probabilistic model of the directed, dynamic process of capability accumulation and product diversification of countries, and show that low- and middle-income countries move from small ecosystem (low complexity) products to large ecosystem (high complexity) products over time. Focusing on cities, Gomez-Lievano and Patterson-Lomba (2018) develop a model for the probability that an individual in a city is employed in a given urban activity which takes into account activity-specific complexity, individual-specific know-how, and city-specific collective know-how. The model is based on complementarity and stochastic accumulation of factors over time, as well as the diversity of factors within urban areas, and is estimated using industry employment data for US cities.

A further set of models aim to connect complexity metrics to well-known economic models for trade and production. Schetter (2021) embeds the ECI framework into a general equilibrium model of international trade. This work shows that a close variant of ECI produces a ranking of countries that corresponds to country complexities derived from a multi-product Eaton and Kortum trade model under an assumption of log-supermodular productivities (e.g., complex economies export relatively more complex products). Bustos and Yildirim (2021) build on Hausmann and Hidalgo (2011) to propose a new approach to approximate the capabilities present in places and industries based on a relaxation of a binary Leontief framework. In their model, a common assumption of nested capabilities (Bustos et al., 2012) and the requirement that all capabilities are present are relaxed. They show that both country- and city-level metrics correlate with income and economic growth, and can predict product appearances and disappearances over time.

The wide variety of approaches introduced above (many of which are implemented using export or employment data) are typically agnostic from a methodological viewpoint on the type of ‘capabilities’ that they capture. Supported by a large literature on the role of locally embedded worker know-how and learning in the capability base of a place (Nelson and Winter, 2002; Neffke and Henning, 2013; Diodato et al., 2018), a parallel set of methods focus on estimating the knowledge complexity of sub-national regions, their occupations and technologies.

Again, we can divide studies into those based on spatial patterns of production and innovation, and those based on alternative modeling strategies. In the first category, focusing on the production of new knowledge, Balland and Rigby (2017) apply the approach developed by Hidalgo and Hausmann (2009) to patent data, using the concentration of patents across technology classes and US cities to compute technology and city complexity metrics. They find that knowledge is unevenly distributed across the United States, and that cities with the most complex technologies are not necessarily those with the highest rates of patenting.

A second strand of literature in this category focuses on occupations. Mealy et al. (2019) apply the approach developed by Hidalgo and Hausmann (2009) to compute complexity metrics based on occupation-region employment concentrations, revealing patterns of occupational specialization across US states. Lo Turco and Maggioni (2021) in this Special Issue deploy information from O*NET on the skill content of jobs. Comparing the complexity of industries (derived from export data using the Hidalgo and Hausmann (2009) approach as above) and the knowledge content of occupations in an industry, they find that complex industries are most intensive in STEM knowledge and skill requirements. The authors propose a new measure of the occupational complexity of industries based on the presence of specific STEM-related skills, and show that this is more predictive of GDP per capita growth for US cities than comparable metrics.

A second category of models focuses on estimating the combinatorial complexity of technologies, made practically possible via the detailed information on technology combinations provided in patent data. Pioneering this approach, Fleming and Sorenson (2001, 2004) developed an evolutionary model of innovation which yields a measure of complexity for individual patents based on the difficulty of combining knowledge subsets (technology sub-classes) in US patent data. This work connects to the NK model (Kauffman and Levin, 1987), a widely used mathematical model which describes evolutionary exploration on a ‘landscape’ and encodes the complexity of a system in terms of its number of elements (N) and their degree of interdependence (K). Extending the approach of Fleming and Sorenson, Broekel (2019) developed a measure of technological complexity via the characterization of the diversity of structural patterns in technologies’ combinatorial networks (which capture the co-occurrence patterns of associated subclasses). By applying this approach to EU patent data, the measure yields an index replicating many features usually associated with technological complexity such as continuous growth over time, spatial concentration and stronger collaboration.

### 2.3 Complexity and performance

The economic complexity literature views economic development as a structural transformation process, whereby economic growth results from the transformation of a country's productive structure from one dominated by simple low-tech activities, typically unprocessed primary products, to one characterized by more technologically advanced manufacturing processes. By emphasizing changes in the composition of output, the complexity approach to growth and development is related to the literature on structural transformation (see Herrendorf et al., 2014 for a review).

This literature emphasizes the difference in productivity levels and growth across sectors and between countries and regions. For example, Duarte and Restuccia (2010) find smaller productivity differences across countries in manufacturing than in agriculture or services, and larger productivity convergence in agriculture and manufacturing than in services. Rodrik (2013) finds unconditional cross-country convergence in manufacturing but not in aggregate productivity. The economic complexity perspective can help us interpret these findings and make them more precise. One implication is that there is productivity convergence at the intensive margin but not at the extensive margin: existing manufacturing activities find it easier to improve, but it is hard to get into new activities because the requisite capabilities may be missing. So, convergence does not occur beyond existing tradable activities. The fact that the product space (see Hidalgo et al., 2007 and the section on relatedness below) is highly structured and that countries are located in different parts of it means that there is significant variance in the capacity to converge based on a country's position in the space. These findings are documented in Felipe et al. (2012), Hausmann et al. (2013), Cristelli et al. (2015), and Gala et al. (2018).

While the link between economic complexity and economic growth has attracted a lot of attention, scholars have also looked at issues of inequality and sustainability. Focusing on the period 1962–2000, Hartmann et al. (2017) find support for the idea that income inequality is lower in countries that export more complex goods. Theoretically, they do not propose a direct connection between the two constructs but rather they suggest that “productive structures represent a high-resolution expression of a number of factors, from institutions to education, that co-evolve with a country's mix of exported products and with the inclusiveness of its economy” (p. 85). Focusing on a different period (1964–2013), Fawaz and Rahnama-Moghadamm (2019) find that trade with economically complex countries is negatively correlated with income inequality. Sbardella et al. (2017) also ran a large-scale cross-country analysis over the period 1963–2008 and found an inverted U shape relationship between an index of complexity and wage inequality, which is in line with the theoretical predictions of Kuznets (1955). Interestingly, however, they also look within the US over the time window 1990–2014 and find an opposite trend, namely that as US counties become more complex, their wage inequality also increases. While the cross-country result is explained by way of social struggles, democratization and institutional strengthening achieved via economic growth, the mechanisms of the within-country result are less neatly explained except that their result might be driven by changes in counties’ sectoral composition. Zhu et al. (2020) also have an interest in understanding the link between the economic complexity of exports and economic inequality at the sub-national level: they focus on the rural-urban divide in Chinese regions (1989–2013) and find that export complexity is associated with lower income inequality only in urban areas where a complex industrial structure offers more diverse working opportunities, greater resilience to shocks, and where workers are more skilled and more capable of networking and increasing their bargaining power with firms. However, they also find that economic inequality does not decline in these regions’ rural areas – a result that they explain by way of limitations imposed on rural inhabitants to move to urban areas. All in all, while increasing economic complexity in countries appears to be associated with greater economic growth and cross-country convergence, its impact on across- and within-country inequality is less clear cut and it deserves more research, especially to pin down the causal mechanisms.

Another important issue has been the connection between economic complexity and environmental performance. Some suggest that economic complexity improves the environmental performance of countries. Sbardella et al. (2018) find a positive relationship between a country's GDP and a measure of green complexity capturing the extent to which a country patents in complex green technologies. Hence, more economically advanced countries are also more sophisticated inventors of green technologies. Another strand of literature shows that low economic inequality comes with greater complexity of green technologies – a result that is explained by the presence of the middle class which creates demand for green innovations and allows for economies of scale in production (Napolitano et al., 2020). Mealy and Teytelboym (2021) in this Special Issue make a significant contribution to the study of green complexity by pooling together all existing environmental goods classifications – from WTO, OECD and APEC - into a unique dataset of green traded products between 1995 and 2014. Using this novel data, they produce a set of country-level indices to measure the extent to which a country is complex in green production capabilities, indices which are also useful to predict countries’ future diversification opportunities.

Others have raised interesting questions about whether richer or more complex economies are also able to curb environmental problems. Romero and Gramkov (2021) find that more economically complex countries have lower greenhouse gas (GHG) emission intensity, and they ascribe this result to the fact that higher complexity leads to more efficient products which are less contaminating. Using a sample of 88 countries observed over the period 2002–2012, Lapatinas et al. (2019) find a positive correlation between a country's economic complexity and its environmental performance, measured as a composite index that includes emissions, protection of health and environmental policies. They also show that it correlates positively with CO2 and PM 2.5 emissions, the latter of which are connected with the emergence of cancer and other health issues. Neagu (2019) finds an inverted U-shape relationship between economic complexity and CO2 emissions in 25 European Union countries over the period 1995–2007.

Finally, while inequality and environmental performance are two issues of concern in the sustainable development agenda, there are other areas that have attracted interest such as migration. If know-how resides in brains, and it moves with difficulty from brain to brain, then moving brains may become critical to the movement of knowhow, the diffusion of technology and the growth of complexity. A long literature has documented the importance of migration in technological diffusion (Saxenian, 2006; Moser et al., 2014; Hausmann and Nedelkoska, 2018; Diodato et al., 2021). Bahar et al. (2014) provide evidence on the international diffusion of competitive advantage, while others have established links between the movement of complex capabilities and FDI flows, suggesting different pathways for countries to build complexity (Khan et al., 2020). Mayneris and Poncet (2015) provide evidence of capability spillovers between co-located foreign and domestic Chinese firms. Coscia et al. (2020) find evidence that business travel from countries with industry knowledge predicts the growth of productivity, employment and exports in those industries in the recipient country, providing further evidence on the importance of human mobility in the movement of specific elements of knowhow. Bahar et al. (2021) in this Special Issue find that more complex economies are characterized by greater immigrants’ birthplace diversity, showing that diversity of workers’ geographical origin – especially among college-educated migrants – leads to greater diversity in the destination export basket. It would be interesting to explore how broader social justice issues, such as social cohesion or human rights protection, change as countries acquire more productive capabilities and become more complex. Are more complex products more sustainable in a social sense or do they require deeper forms of human cooperation that are correlated with greater social cohesion and rights?

### 2.4 Complexity and geographical scale

Complex economic activities tend to concentrate in space. Hidalgo and Hausmann (2009) exploit this fact in their measures of complexity at the international scale. Interest in economic complexity, and its relevance for understanding uneven development, has grown just as rapidly at the sub-national scale. In early work using export data for a panel of Chinese cities, Poncet and Waldemar (2013) report that economic complexity is a robust determinant of economic growth. Using patent data, Balland and Rigby (2017) explore shifts in the complexity of knowledge production across US metropolitan areas. For EU regions, Balland et al. (2019) use measures of relatedness density to identify the knowledge cores of regions and to proxy for the cost of developing new technology growth paths. The returns to developing these growth paths are measured with complexity-based indicators of the value of competing technologies. Gao and Zhou (2018) explore the evolution of complexity across Chinese provinces since 2000. They report faster growth in complexity for coastal regions of China and a strong positive relationship between economic complexity and the level of GDP per capita. Firm-level export data is used by Zhou et al. (2019) to document the increase in complexity of Chinese exports at the city-level. Export upgrading is tied to capabilities that are sourced within the firm and within the region.

In the 2021 Research Policy Special Issue on Economic Complexity, several papers confirm that complex knowledge is unevenly distributed across European regions. Antonelli et al. (2021) provide evidence that complex knowledge concentrates in the most productive European regions. They report that the complexity of the regional knowledge base affects regional innovation positively, but productivity performance negatively. Using similar metrics, Pintar and Scherngell (2021) show that the most complex technologies, such as Digital Communication, Telecommunications, or Computer Technology tend to be the most spatially concentrated, i.e., the least ubiquitous. Mewes and Broekel (2021) find a similar pattern using a different approach to complexity based on the structural diversity of knowledge development Broekel (2019). They find that the ability of regions to produce and exploit complex technologies explains regional economic growth. All these papers stress the fundamental idea that complexity metrics, of one form or another, allow the authors to go beyond patent counts and capture qualitative differences in regional knowledge production. Pintar and Scherngell (2021), in particular, highlight why and how this can benefit the development of place-based innovation policy such as the smart specialization strategy.

As noted above, complex economic activities cluster in space. However, they do not cluster in random parts of the world. O'Clery et al. (2018) and Balland et al. (2020) reveal that complex economic activities systematically concentrate in large cities. Complexity, alone, can explain from 40% to 80% of the variance in urban concentration of occupations, industries, scientific fields, and technologies. The spatial concentration of cutting-edge technologies in large cities has increased since 1850. As discussed in Section 2.1, complex activities require a deeper division of knowledge. This division of knowledge creates high coordination costs that cities help solve by creating multiple mixing and matching opportunities. Gomez-Lievano et al. (2016) contribute to this literature by developing a model that explains why complex economic activities tend to occur in larger and more diverse urban hubs. Frank et al. (2018) show that this spatial variance expands beyond the production of knowledge. The cities that face a greater impact of automation on employment are the smaller, least complex and least diversified ones, adding another dynamic source of spatial inequality going forward.

### 2.5 Relatedness

Relatedness, which describes the relationship between different classes of economic activities, has emerged as a powerful concept to explain economic diversification and technological upgrading (Breschi et al., 2003; Hidalgo et al., 2007; Neffke et al., 2011). There are two roots with different interpretations of the underpinnings of relatedness. On the one hand, two activities are related if their vectors of capability requirements (the **P***_pa_*) are similar. As a consequence, it is easier for a country to move from existing activities to related activities because it involves fewer missing capabilities. In this interpretation, moving towards related activities is more feasible, but not necessarily more valuable.

The alternative, and older, approach to relatedness is rooted in learning processes and encompasses multiple components, alongside the core concepts of complementarity and similarity. References to complementarity and coherence first surged with the emergence of a resource-based view of the firm highlighting that competitive advantage arises from combining heterogeneous resources (Barney, 1991, Dyer and Singh, 1998, Teece et al., 1994). The concept of similarity emphasizes that shared cognitive features and a joint root in the same knowledge domains facilitates more effective and easier learning (Stuart, 1998). Nooteboom (1999) added that the potential gains from combining similar (knowledge) resources peak at an optimal level of cognitive distance (relatedness), as too small and large dissimilarities either hamper learning or offer too little potential for novelty.

Crucially, while relatedness describes the proximity between different categories of economic activities (or sets thereof), its application usually requires a more structural perspective. A key substantive focus within the research area of relatedness is the transformation of economies through the mechanism of diversification. The principle of relatedness stresses that the probability of diversification is shaped by the existence of multiple related activities, and not by any one particular activity. Assessing activities based on their embeddedness within a wider set of relations to other activities underlies prominent concepts such as corporate coherence (Teece et al., 1994) and related as well as unrelated variety (Frenken et al., 2007). The literature on (resource) completeness and on (resource) portfolios add in this context that the relation between two activities is frequently shaped by the presence of expertise in (multiple) others (Carnahan et al., 2010; Medcof, 2000).

Consequently, to understand and apply the idea of relatedness, it is essential to look at the full set of relations between activities. This set is frequently defined, and often mapped, as a relatedness space that visualizes different sorts of proximities. Since the original product space mapping set out by Hidalgo et al. (2007), we now regularly refer to the knowledge space built around technologies (Breschi et al., 2003; Kogler et al., 2013), the industry space (Essletzbichler, 2015; Neffke et al., 2011), the occupation space (Muneepeerakul et al., 2013), and the space of scientific disciplines (Lyu et al., 2020). Measuring the proximity of subsets of economic activities deploying ‘space’ or ‘network’ perspectives and generalizing it to the principle of relatedness (Hidalgo et al., 2018) has massively contributed to the popularity of the relatedness concept within and outside academia. In particular, it gave rise to a new perspective on economic development by providing a methodological toolbox to identify path dependencies in the diversification processes of countries, regions, and firms (Breschi et al., 2003; Hidalgo et al., 2007; Neffke et al., 2011).

Since its inception, relatedness has been measured at the “phenotypic” level (i.e., the **M***_cp_* matrix is used to observe the co-occurrence of products and infer which ones are similar), without knowledge of the “genotypic” level (which presumes knowledge of **P***_pa_*). Most studies on relatedness tell us which products are related but they do not tell us why. Several papers in the past years have begun to change that. Diodato et al. (2018) build on Ellison et al. (2010) to show that co-location of industries is driven, increasingly but not uniquely, by industries sharing occupational needs. Similarly, Diodato and Schetter (2020) show that a country's industrial diversification path can be predicted by using a product space calculated from the similarity in the occupational requirements of products in the **P***_pa_*, assuming that the inputs are occupations. Using patent data, Juhász et al. (2021) show that the development of technological relatedness is shaped by the co-concentration of technological capabilities in space. Pugliese et al. (2019) tackle the same question, albeit with a different method: they formalize the concept of multi-layer space: instead of looking at industry similarities based on an underlying extra layer (e.g. occupational overlap of industries as in Diodato and Schetter, 2020), they look at the similarity between different categories of data, by measuring the co-occurrence of different layers (e.g. similarity between a patent class and an industry). Their multi-layer product space is a weighted version of the following formula: **MPS***_pa_* = **M***_cp_*^T^ **C**_*ca*_.

In this Special Issue, Catalán et al. (2021) expand on Pugliese et al. (2019). The authors combine information on scientific (publications) and technological (patents) relatedness to develop broader measures of the competences of countries based on the cross-density of scientific and technological capabilities. While this measure does not explain technological diversification better than traditional unidimensional measures, the paper advances a promising new research trajectory exploring the multi-dimensional nature of relatedness.

More research is also needed to better understand the path-dependent nature of relatedness processes and how this translates into comparative advantage and growth. Hausmann et al. (2021) in this Special Issue focus on these matters. By developing a modified Ricardian-model, the authors estimate the comparative advantage of an industry in a specific location based on the comparative advantage of similar industries and similar locations. It is shown that this approach predicts long-term employment growth. In addition, the paper overcomes a shortcoming of many traditional trade models that treat industries as independent. In contrast, the proposed model allows industry productivity to be impacted by those of related industries.

Dosi et al. (2021), in this Special Issue, continue the tradition of the early literature on relatedness by evaluating the role of corporate coherence (Teece et al., 1994). Besides adding much-needed empirical evidence in the context of developing countries, the authors also explicitly address and account for firms’ heterogeneity before the diversification event. Crucially, the authors show that coherence not only matters for firms’ growth, but also for their profitability. Yet, the relevance of relatedness for economic performance is still anything but clear. So far, the main mechanism whereby relatedness shapes economic performance is the path-dependent selection process guiding (some) firms and regions towards activities with higher economic potential (Hidalgo et al., 2007). This process, however, cannot be fully understood from a relatedness perspective alone. Using trade data, Pinheiro et al. (2021), in this Special Issue, dig into the relationship between unrelated diversification and complexity. The study shows that the direction of diversification, related (path-dependent) or unrelated (path-breaking), is conditional on the existing level of country complexity. In particular, unrelated diversification becomes more likely at high levels of complexity. In summary, the studies in the 2021 Research Policy Special Issue on Economic Complexity clearly highlight the need to move beyond investigating relatedness in isolation from other factors. This is particularly the case as relatedness provides indications about the direction and speed of path-dependent processes but is insufficient to evaluate these processes' economic value. The latter is rather shaped by the interplay of relatedness and complexity, which possibly represents the most promising avenue for further research.

### 2.6 Division of knowledge

The division of knowledge has served as the backbone of the main research themes in the literature: the complexity of production, relatedness and diversification. However, there are more nuanced aspects of this paradigm that are increasingly becoming the subject of research in the literature.

First, how does trade change the picture? In autarchy, there is little doubt about the links between individual specialization, societal diversification, and growth. But in an open economy, market pressure surely must be pushing against diversification: even a country that is more productive in all industries would find it convenient to disinvest from some activities (Dornbusch et al., 1977). This should happen because resources are finite and employing them in one industry has opportunity costs. A handful of papers are beginning to investigate how the paradigm of economic complexity is affected by trade. Schetter (2021) for instance shows that if technology is log-supermodular, then trade will lead to specialization, but (less) complex countries will tend to specialize in (less) complex goods. Schetter shows that, in such a world, the economic complexity index would be an appropriate measure of a country's technological advantage.

This suggests that growing economies can continue to diversify in terms of know-how and productive capabilities but they choose to use these capabilities in fewer, more complex products, i.e., products that require more knowhow. In this Special Issue Van Dam and Frenken (2021) propose a combinatorial model of growth that, although not in general equilibrium, incorporates an opportunity cost mechanism (interpreted as rising salaries in the growing country). They show that a country diversifies only up to a certain point, when it begins abandoning simpler varieties. This can also account for the empirical observation that at a high level of development, the production portfolio of countries tends to (moderately) re-concentrate (Imbs and Wacziarg, 2003; Cadot et al., 2011). Schetter (2020) shows that if one allows for differences in quality, advanced countries can remain in low complexity products by specializing in higher qualities.

Second, as noted by Becker and Murphy (1992), specialization and division of knowledge necessarily come with coordination costs. This aspect is certainly understudied in the context of the literature in economic complexity, but there are a few papers that explore this new domain. Neffke (2019), for instance, shows that the benefits of the division of labor only materialize if specialized individuals are embedded into teams of complementary workers. In this Special Issue, Botta et al. (2021) compare the functioning of financial markets to the principles of economic complexity and argue that, much like for production, financial knowledge is distributed across many specialized agents. They find that this type of complexity is not associated with positive economic growth but increases its volatility.

Finally, technology is not just about tacit knowledge or know-how. It is also the knowledge embodied in tools (e.g., machines) and codes (e.g., blueprints). When technological progress enables us to transfer human know-how into autonomous machines, the expertise of some workers may become obsolete. While studying the effects of automation has a long tradition in economics (Autor et al., 2003; Acemoglu and Restrepo, 2020), the literature on economic complexity is now beginning to explore the meaning of automation from its particular vantage point. Much like the product space can predict the evolution of an economy in its growth trajectory, a few recent papers use information on the knowledge, skills and abilities required by different occupations to construct occupational spaces and explore the impact of automation on labor market outcomes. Alabdulkareem et al. (2018), for instance, use cluster analysis to show that wage polarization in the labor market is reflected in a polarized network of skills, with low-paying jobs belonging to an isolated community of sensory-physical abilities. Nedelkoska et al. (2018) develop task and occupation spaces using knowledge, skills and abilities from O*NET and find that knowledge is highly transferable across tasks, but occupations that are at high risk of automation all belong to parts of the occupational space that make the necessary switch harder to achieve. Del Rio-Chanona et al. (2019) combine a search and matching model with occupational mobility networks and argue that the latter is key to understanding long-term dynamics of unemployment after a technological transition.

Overall, the role of human capital is now a vibrant area of research in the literature. Hence, the division of knowledge may not only represent the origin of economic complexity, but also its future.

## 3 Conclusion: economic complexity and innovation policy

Since the first industrial revolution, exponential gains in economic complexity have accrued in tandem with unprecedented levels of innovation and wealth generation. While a trillion isolated individuals could never build an airplane, let alone put a human on the moon, a far smaller number of interacting agents who specialize and trade can, given the right incentives, produce a dizzying array of massively complex products. Complex products, and the complex sets of capabilities on which they rest, emerge from deep divisions of labor driven by competition within the market economy. This is why the first direct policy implication of the field of economic complexity has been for countries and regions to specialize into more complex economic activities (Hausmann et al., 2011; Balland et al., 2019; Hidalgo, 2021). Supporting economic upgrading by building complex capabilities is a superior development strategy to chasing the ability to produce high-priced goods. Commodity prices can shift rapidly with changing market conditions, regulations, and customer preferences. Developing the capabilities to create and produce complex products is a viable path to secure long-run growth as these capabilities tend to evolve in self-reinforcing processes of recombination, rewarding most of those actors, firms, and places that are already embedded in networks of complex activity.

One unfortunate consequence of the growth and concentration of economic complexity is rising levels of inequality. By their nature, more complex systems also tend to be more unequal. Preferential attachment, compounding, self-reinforcing feedback loops, and multiplicative processes that are inherent to complex adaptive systems increase inequality. As a result, some individuals, organizations, and places will occupy privileged positions in which they can leverage larger parts of an economy's structure and accumulate most of the benefits. Others will be much less fortunate, either because they have the wrong skills, they are located in the wrong place, or they face other factors that prevent them from engaging in economic activities. In our modern world, inequality is striking. Walmart's Walton family leverages labor structures, Warren Buffet leverages capital, and digital platforms leverage code and media to secure and expand dominant economic positions. This is consistent with earlier innovation research which points at the uneven distributional effects of technological revolutions or advancements (e.g., Perez, 2013) and suggests a need to find new ways to govern capitalism in order to avoid the concentration of economic and political power to the detriment of society (Giuliani, 2018). These ideas echo recent empirical research showing how technological giants handling and generating highly complex knowledge in domains such as ICT and artificial intelligence have built knowledge monopolies from innovations and exclusive access to data (Rikap and Lundvall, 2020), while others have raised concerns about the geographic distributional imbalances caused by high tech companies’ concentration of monopoly rents (Feldman et al., 2021). These monopolies create fat-tailed distributions and challenge existing (cohesion-oriented) social structures. The imbalances and inequalities generated by growing complexity require new policy responses to generate equitable returns and secure futures for all.

As complexity shapes multiple dimensions of economic development, the inequality that it generates is also manifest in different forms. One of the most pervasive of those forms is uneven spatial development. Larger, urban centers in advanced industrialized economies are the primary beneficiaries of the growth of complexity (Mewes and Broekel, 2021, Pintar and Scherngell, 2021, Van Dam and Frenken, 2021). Given the self-reinforcing nature of complexity, uneven development is difficult to manage or slow down. Yet, policy must confront the spatial consequences of complexity, rapid urbanization and the siphoning of resources from peripheral regions that are often viewed as “places that don't matter”, fueling populism and social unrest (Rodríguez-Pose, 2018). Given the path-dependent nature of these processes, it is more than likely that complexity dynamics will tend to reinforce the current status rather than breaking-up existing structures. In any case, the fundamental dynamics of economic complexity require a policy response.

How do we raise prospects for those working in less complex economies? A general goal is to help these economies leverage the capabilities that they already possess to diversify into more complex forms of economic activity. Thus, smart investment for economic development involves identifying the foundations of existing strengths and mapping potential pathways towards a more complex set of capabilities. Identification of these pathways requires knowledge of the “distances” between economic activities. Measures of economic relatedness that span occupations, products, industries, and technologies provide these “distance” metrics (see Pinheiro et al., 2021 in this Special Issue). This second complexity policy directive stems from the fact that new sets of capabilities are created by recombining pre-existing capabilities (Mowery et al., 1998). The toolbox of economic complexity is, in fact, also becoming more prominent in policy programs of the European Commission, including innovation and industrial strategies (Pugliese and Tacchella, 2020) and smart specialization (Balland et al., 2019). Yet, while relatedness and complexity seem to be considered in most support strategies, it is rare to find them strategically combined, as regional priorities seem to be based on complexity or relatedness concerns in isolation (Deegan et al., 2021). Further research is required to develop place-based policy interventions that utilize relatedness to support the emergence of more complex activities.

Continued growth in levels of economic complexity implies further specialization that is likely to demand increased interdependencies in the economic system. This has important consequences for questions of resilience to exogenous shocks at multiple spatial scales. While more interdependent systems can be more vulnerable to sudden disruptions, complex systems in contrast usually exhibit remarkable robustness to this. In this case, the increasing interdependence in the system needs to go hand-in-hand with decentralization of decision making and capabilities, as well as dynamic capabilities of reconfiguration. The latter may be as much about structure as about agency. Here, research has just started. The same applies to sustainability since enhanced complexity in some regions of the world may have a neither neutral nor positive impact on other non-complex regions. Increasing interactions of specialized actors are likely to imply more movements of people, goods, and energy, all of which involve substantial environmental and social costs. For instance, increasing mobility of talent not only drains environmental resources, but also challenges established social networks and cultural embeddedness, and as a consequence individual as well as social well-being.

The growing complexity of the economy has yielded levels of prosperity and innovation that would not have been imaginable only a few decades ago. It has provided policy opportunities for economic development but also an entirely new set of challenges. Managing human hyperconnectivity and its consequences – ranging from climate change, inequality, spatial polarization, and disease transmission – might be one of the most pressing policy challenges of the 21st century.

## Credits

All authors have participated in drafting the article, revising it critically for important intellectual content, and approval of the final version.

## Declaration of Competing Interest

The authors have no affiliation with any organization with a direct or indirect financial interest in the subject matter discussed in the manuscript.

## Footnotes

1 The distinction between codified knowledge and tacit knowledge is related to the philosophical debate between Karl Popper and Michael Polanyi. Popper emphasized what he called objective knowledge, as something that exists in ideas that are out in society and that evolve in a social process as they are challenged and falsified. Michael Polanyi, in reaction, added that there was an important dimension of knowledge - what he called personal knowledge - that was not out there, but in the person and was not conceptual and hence, not expressible in words or codes. See Popper (1972) and Polanyi (1958). Note, additionally, that what is *codifiable* changes with technology: AI for instance is managing to embed, into tools and codes, know-how that was previously thought to be uniquely human. In Section 2.6, we discuss how codification of know-how is an emerging area of investigation for economic complexity research.

2 The expression *extensive margin*, which is commonly used in economics, refers to the variety of the object under discussion (the variety of know-how in this case). This is in contrast to the *intensive margin*, which refers to its amount. A process of growth at the extensive margin is described, for instance, in Jacobs (1969), where she notes that our ancestors did not expand their economies by accumulating more “wild seeds and nuts”, but by adding new kinds of work. Solow's model, in contrast, provides an example of a growth process at the intensive margin, that is through capital accumulation.

3 A poverty trap exists when vicious cycles of causation keep some countries underdeveloped. The concept is typically associated with the presence of multiple equilibria: when a country manages to escape the trap, the cycle of causation may become virtuous and lead to sustained economic growth. Hausmann and Hidalgo (2011) call this particular form of poverty trap – where combinatorial possibilities give incentives to acquire new elements, only if you already have many elements – a *quiescence trap.*

4 For instance, Hill (1973) developed a method to rank locations that is very similar to the Economic Complexity Index. Moreover, the concept of nestedness – developed by Patterson & Atmar (1986), Atmar & Patterson (1993), Almeida et al. (2008) to study the location of species – is applicable to the kinds of matrices used in economic complexity, as shown in Bustos et al. (2012).

5 Dimensionality reduction is the process of transforming large, multi-dimensional datasets into more compact information, easier to process by both humans and computers. Techniques are typically designed to maintain, in the transformed data, some desired property of the original ones.

6 These theories use increasing returns associated with the fixed cost of developing new varieties. They use Dixit-Stiglitz production functions where all inputs enter with equal rates of substitution. And they use monopolistic competition in order to create an equilibrium in spite of the convexity in the production function. Under these conditions, productivity is related to the number of inputs used.

7 Hausmann et al. (2021) in this Special Issue justify these conclusions in a Ricardian framework.

## References

[bib0001] Acemoglu D., Restrepo P. (2020). Robots and jobs: evidence from US labor markets. J. Polit. Econ..

[bib0002] Aghion P., Howitt P. (1992). A model of growth through creative destruction. Econometrica.

[bib0003] Alabdulkareem A., Frank M.R., Sun L., AlShebli B., Hidalgo C., Rahwan I. (2018). Unpacking the polarization of workplace skills. Sci Adv.

[bib0004] Almeida-Neto M., Guimaraes P., Guimaraes P.R., Loyola R.D., Ulrich W. (2008). A consistent metric for nestedness analysis in ecological systems: reconciling concept and measurement. Oikos.

[bib0005] Antonelli C., Crespi F., Quatraro F. (2021). Knowledge complexity and the mechanisms of knowledge generation and exploitation: the European evidence. Res. Policy.

[bib0006] Atkin, D., Costinot, A., and Fukui, M. (2021) Globalization and the ladder of development: Pushed to the top or held at the bottom? [paper]. Retrieved from https://masaofukui.com/wp-content/uploads/2021/09/mutvc_LATEST.pdf.

[bib0007] Atmar W., Patterson B.D. (1993). The measure of order and disorder in the distribution of species in fragmented habitat. Oecologia.

[bib0008] Autor D.H., Levy F., Murnane R.J. (2003). The skill content of recent technological change: an empirical exploration. Q. J. Econ..

[bib0009] Bahar D., Hausmann R., Hidalgo C.A. (2014). Neighbors and the evolution of the comparative advantage of nations: evidence of international knowledge diffusion?. J. Int. Econ..

[bib0010] Bahar D., Rapoport H., Turati F. (2021). Does birthplace diversity affect economic complexity? Crosscountry Evidence. Res. Policy.

[bib0011] Balland P.A., Rigby D. (2017). The geography of complex knowledge. Econ. Geogr..

[bib0012] Balland P.A., Boschma R., Crespo J., Rigby D.L. (2019). Smart specialization policy in the European Union: relatedness, knowledge complexity and regional diversification. Reg. Stud..

[bib0013] Balland P.A., Jara-Figueroa C., Petralia S.G., Steijn M.P., Rigby D.L., Hidalgo C.A. (2020). Complex economic activities concentrate in large cities. Nat. Hum. Behav..

[bib0014] Barney J. (1991). Firm resources and sustained competitive advantage. J. Manage..

[bib0015] Becker G.S., Murphy K.M. (1992). The division of labor, coordination costs, and knowledge. Q. J. Econ..

[bib0016] Bettencourt L.M., Samaniego H., Youn H. (2014). Professional diversity and the productivity of cities. Sci. Rep..

[bib0017] Botta A., Caverzasi E., Russo A. (2021). When complexity meets finance: a contribution to the study of the macroeconomic effects of complex financial systems. Res. Policy.

[bib0018] Breschi S., Lissoni F., Malerba F. (2003). Knowledge-relatedness in firm technological diversification. Res. Policy.

[bib0019] Broekel T. (2019). Using structural diversity to measure the complexity of technologies. PLoS One.

[bib0020] Brummitt C.D., Gómez-Liévano A., Hausmann R., Bonds M.H. (2020). Machine-learned patterns suggest that diversification drives economic development. J. R. Soc., Interface.

[bib0021] Bustos S., Gomez C., Hausmann R., Hidalgo C.A. (2012). The dynamics of nestedness predicts the evolution of industrial ecosystems. PLoS One.

[bib0022] Bustos S., Yıldırım M.A. (2021). Production ability and economic growth. Res. Policy.

[bib0023] Cadot O., Carrère C., Strauss-Kahn V. (2011). Export diversification: what's behind the hump?. Rev. Econ. Stat..

[bib0024] Carnahan S., Agarwal R., Campbell B. (2010). The effect of firm compensation structures on the mobility and entrepreneurship of extreme performers. Business.

[bib0025] Catalán P., Navarrete C., Figueroa F. (2021). The scientific and technological cross-space: is technological diversification driven by scientific endogenous capacity?. Res. Policy.

[bib0026] Cristelli M., Gabrielli A., Tacchella A., Caldarelli G., Pietronero L. (2013). Measuring the intangibles: a metrics for the economic complexity of countries and products. PLoS One.

[bib0027] Cristelli M., Tacchella A., Pietronero L. (2015). The heterogeneous dynamics of economic complexity. PLoS One.

[bib0028] Coscia M., Neffke F.M., Hausmann R. (2020). Knowledge diffusion in the network of international business travel. Nat. Hum. Behav..

[bib0029] Deegan J., Broekel T., Fitjar R.D. (2021). Searching through the Haystack: the relatedness and complexity of priorities in smart specialization strategies. Econ Geogr.

[bib0030] del Rio-Chanona R.M., Mealy P., Beguerisse-Díaz M., Lafond F., Farmer J.D. (2019). https://arxiv.org/pdf/1906.04086.

[bib0032] Diodato D., Morrison A., Petralia S. (2021). Migration and invention in the Age of Mass Migration. J. Econ. Geogr..

[bib0033] Diodato D., Neffke F., O'Clery N (2018). Why do industries coagglomerate? How Marshallian externalities differ by industry and have evolved over time. J. Urban Econ..

[bib0034] Diodato D., Schetter U. (2020). Pathways to prosperity: economic development with a network of industries. Mimeo.

[bib0035] Dornbusch R., Fischer S., Samuelson P.A. (1977). Comparative advantage, trade, and payments in a Ricardian model with a continuum of goods. Am. Econ. Rev..

[bib0036] Dosi G., Mathew N., Pugliese E. (2021). What a firm produces matters: processes of diversification, coherence and performances of Indian manufacturing firms. Res. Policy.

[bib0037] Duarte M., Restuccia D. (2010). The role of the structural transformation in aggregate productivity. Q. J. Econ..

[bib0038] Dyer J.H., Singh H. (1998). The relational view: cooperative strategy and sources of interorganizational competitive advantage. Acad. Manage. Rev..

[bib0039] Eaton J., Kortum S. (2002). Technology, geography, and trade. Econometrica.

[bib0040] Ellison G., Glaeser E.L., Kerr W.R. (2010). What causes industry agglomeration? Evidence from coagglomeration patterns. Am. Econ. Rev..

[bib0041] Essletzbichler J. (2015). Relatedness, industrial branching and technological cohesion in US Metropolitan Areas. Reg. Stud..

[bib0042] Fawaz F., Rahnama-Moghadamm M. (2019). Spatial dependence of global income inequality: the role of economic complexity. Int. Trade J..

[bib0043] Feldman M., Guy F., Iammarino S. (2021). Regional income disparities, monopoly and finance. Cambridge J. Regions, Econ. Soc..

[bib0044] Felipe J., Kumar U., Abdon A., Bacate M. (2012). Product complexity and economic development. Struct. Change Econ. Dynamics.

[bib0045] Fleming L., Sorenson O. (2001). Technology as a complex adaptive system: evidence from patent data. Res. Policy.

[bib0046] Fleming L., Sorenson O. (2004). Science as a map in technological search. Strat. Manage. J..

[bib0047] Frank M.R., Sun L., Cebrian M., Youn H., Rahwan I. (2018). Small cities face greater impact from automation. J. R. Soc., Interface.

[bib0048] Frenken K., Van Oort F., Verburg T. (2007). Related variety, unrelated variety and regional economic growth. Reg. Stud..

[bib0049] Gala P., Rocha I.L., Magacho G.R. (2018). The structuralist revenge: economic complexity as an important dimension to evaluate growth and development. Brazilian J. Polit. Econ..

[bib0050] Gao J., Zhou T. (2018). Quantifying China's regional economic complexity. Physica A.

[bib0051] Giuliani E. (2018). Regulating global capitalism amid rampant corporate wrongdoing – Reply to “Three frames for innovation policy”,. Res. Policy.

[bib0052] Gomez-Lievano A., Patterson-Lomba O., Hausmann R. (2016). Explaining the prevalence, scaling and variance of urban phenomena. Nat. Human Behav..

[bib0053] Gomez-Lievano A., Patterson-Lomba O. (2018). https://arxiv.org/pdf/1812.02842.

[bib0054] Grossman G.M., Helpman E. (1991). Trade, knowledge spillovers, and growth. Eur. Econ. Rev..

[bib0055] Hartmann D., Guevara M.R., Jara-Figueroa C., Aristarán M., Hidalgo C.A. (2017). Linking economic complexity, institutions, and income inequality. World Dev.

[bib0056] Hausmann R. (2013). https://www.project-syndicate.org/.

[bib0057] Hausmann R., Hidalgo C.A. (2011). The network structure of economic output. J. Econ. Growth.

[bib0058] Hausmann R., Hidalgo C.A., Bustos S., Coscia M., Simoes A. (2014).

[bib0059] Hausmann R., Nedelkoska L. (2018). Welcome home in a crisis: effects of return migration on the non-migrants' wages and employment. Eur. Econ. Rev..

[bib0060] Hausmann R., Stock D.P., Yıldırım M.A. (2021). Implied comparative advantage. Res. Policy.

[bib0061] Herrendorf B., Rogerson R., Valentinyi A. (2014).

[bib0062] Hill M.O. (1973). Reciprocal averaging: an eigenvector method of ordination. J. Ecol..

[bib0063] Hidalgo C.A. (2021). Economic complexity theory and applications. Nat. Rev. Phys..

[bib0064] Hidalgo C.A., Hausmann R. (2009). The building blocks of economic complexity. Proc. Natl. Acad. Sci. USA.

[bib0065] Hidalgo C.A., Klinger B., Barabási A.L., Hausmann R. (2007). The product space conditions the development of nations. Science.

[bib0066] Hidalgo C.A., Balland P.A., Boschma R., Delgado M., Feldman M., Frenken K., Glaeser E., Canfei H., Kogler D.F., Morrison A., Neffke F., Rigby D., Stern S., Zheng S., Zhu S. (2018). International Conference on Complex Systems.

[bib0067] Hulten, C.R., (2001). Total factor productivity: a short biography, in Hulten, C. R., Dean, E. R., Harper, M. J. (eds.) *New Developments in Productivity Analysis*, Charles R. Hulten, Edwin R. Dean, and Michael J. Harper, eds., Studies in Income and Wealth, vol. 63, The University of Chicago Press For the National Bureau of Economic Research, Chicago, 2001, 1–47.

[bib0068] Imbs J., Wacziarg R. (2003). Stages of diversification. Am. Econ. Rev..

[bib0069] Jacobs J. (1969).

[bib0070] Jones B.F. (2009). The burden of knowledge and the “death of the renaissance man”: is innovation getting harder?. Rev. Econ. Stud..

[bib0071] Juhász S., Broekel T., Boschma R. (2021). Explaining dynamics of relatedness: the role of co-location, complexity and collaboration. Papers Region. Sci..

[bib0072] Kahneman D. (2011).

[bib0073] Kauffman S., Levin S. (1987). Towards a general theory of adaptive walks on rugged landscapes. J. Theor. Biol..

[bib0074] Khan H., Khan H., Khan M.A. (2020). Causal nexus between economic complexity and FDI: empirical evidence from time series analysis. Chinese Econ..

[bib0075] Kogler D.F., Rigby D.L., Tucker I. (2013). Mapping knowledge space and technological relatedness in US cities. European Plann. Studies.

[bib0076] Kuznets S. (1955). Economic growth and income inequality. Am. Econ. Rev..

[bib0077] Lapatinas A., Garas A., Boleti E., Kyriakou A. (2019). https://link.springer.com/article/10.1007/s00181-018-1511-y.

[bib0078] Lo Turco A., Maggioni D. (2021). The knowledge and skill content of production complexity. Res Policy.

[bib0079] Lyu X., Zhou P., Leydesdorff L. (2020). Eco-systems mapping and forecasting of techno-science linkages at the level of scholarly journals and fields. Scientometrics.

[bib0080] Mayneris F., Poncet S. (2015). Chinese firms' entry to export markets: the role of foreign export spillovers. World Bank Econ. Rev..

[bib0081] Mealy P., Farmer J.D., Teytelboym A. (2019). Interpreting economic complexity. Sci. Adv..

[bib0082] Mealy P., Teytelboym A. (2021). Economic complexity and the green economy. Res. Policy.

[bib0083] Medcof J.W. (2000). The resource-based view and transnational technology strategy. J. High Technol. Manage. Res..

[bib130] Mewes L., Broekel T. (2021). Technological complexity and economic growth of regions. Res. Policy.

[bib0085] Morrison G., Buldyrev S.V., Imbruno M., Arrieta O.A.D., Rungi A., Riccaboni M., Pammolli F. (2017). On economic complexity and the fitness of nations. Sci. Rep..

[bib0086] Moser P., Voena A., Waldinger F. (2014). German Jewish émigrés and US invention. Am. Econ. Rev..

[bib0087] Mowery D.C., Oxley J.E., Silverman B.S. (1998). Technological overlap and interfirm cooperation: implications for the resource-based view of the firm. Res. Policy.

[bib0088] Muneepeerakul R., Lobo J., Shutters S.T., Goméz-Liévano A., Qubbaj M.R. (2013). Urban economies and occupation space: can they get “there” from “here”?. PLoS One.

[bib0089] Napolitano, L., Consoli, D., Barbieri, N., and Perruchas, F. (2020). Green innovation and income inequality: a complex system analysis [SPRU working paper series]. Retrieved from https://papers.ssrn.com/sol3/papers.cfm?abstract_id=3663871. 10.1016/j.strueco.2022.09.007 PMC9733491 36518901

[bib0090] Neagu O. (2019). The link between economic complexity and carbon emissions in the European Union countries: a model based on the environmental Kuznets Curve (EKC) approach. Sustainability.

[bib0091] Nedelkoska, L., Diodato, D., and Neffke, F. (2018). Is our human capital general enough to withstand the current wave of technological change? [CID Research Fellow and Graduate Student Working Paper No. 93]. Retrieved from https://dash.harvard.edu/bitstream/handle/1/37366813/93.pdf?sequence=1andisAllowed=y.

[bib0092] Neffke F. (2019). The value of complementary co-workers. Sci Adv.

[bib0093] Neffke F., Henning M., Boschma R. (2011). How do regions diversify over time? Industry relatedness and the Development of new growth paths in regions. Econ. Geogr..

[bib0094] Neffke F., Henning M. (2013). Skill relatedness and firm diversification. S*trat. Manage. J.*.

[bib0095] Nelson R.R., Winter S.G. (2002). Evolutionary theorizing in economics. J. Econ. Perspect..

[bib0096] Nooteboom B. (1999). Innovation, learning and industrial organisation. Cambridge J. Econ..

[bib0097] O'Clery, N., Chaparro, J.C., Gomez-Lievano, A., and Lora, E. (2018). *Skill Diversity As the Foundation of Formal Employment Creation in Cities*. Technical report, Working Paper at Center for International Development at Harvard University.

[bib0098] O'Clery N., Yıldırım M.A., Hausmann R. (2021). Productive ecosystems and the arrow of development. Nat. Commun..

[bib0099] Patterson B.D., Atmar W. (1986). Nested subsets and the structure of insular mammalian faunas and archipelagos. Biol. J. Linn. Soc..

[bib0100] Perez C., Fagerberg J., Martin B.R., Andersen E.S. (2013). Innovation Studies: Evolution and Future Challenges.

[bib0101] Petralia S., Balland P.A., Morrison A. (2017). Climbing the ladder of technological development. Res. Policy.

[bib0102] Pinheiro F.L., Hartmann D., Boschma R., Hidalgo C.A. (2021). The time and frequency of unrelated diversification. Res. Policy.

[bib0103] Pintar N., Scherngell T. (2021). The complex nature of regional knowledge production: evidence on European regions. Res. Policy.

[bib0104] Polanyi M. (1958).

[bib0105] Popper K. (1972).

[bib0106] Poncet S., De Waldemar F.S. (2013). Export upgrading and growth: the prerequisite of domestic embeddedness. World Dev..

[bib0108] Pritchett L. (1997). Divergence, big time. J. Econ. Perspect..

[bib0107] Pugliese E., Cimini G., Patelli A., Zaccaria A., Pietronero L., Gabrielli A. (2019). Unfolding the innovation system for the development of countries: coevolution of science, technology and production. Sci. Rep..

[bib0109] Pugliese, E., and Tacchella, A., (2020) Economic Complexity for competitiveness and innovation: a novel bottom-up strategy linking global and regional capacities [paper]. Retrieved from https://econpapers.repec.org/paper/iptiptwpa/jrc122086.htm.

[bib0110] Rikap C., Lundvall B.Å. (2020). Big tech, knowledge predation and the implications for development. Innov. Develop..

[bib0111] Rodríguez-Pose A. (2018). The revenge of the places that don't matter (and what to do about it). Cambridge J. Regions, Econ. Soc..

[bib0112] Rodrik D. (2013). Unconditional convergence in manufacturing. Q. J. Econ..

[bib0113] Romero J.P., Gramkow C. (2021). Economic complexity and greenhouse gas emissions. World Dev..

[bib0114] Saxenian, A. (2006). International Mobility of Engineers and the Rise of Entrepreneurship in the Periphery [paper]. Retrieved from https://www.econstor.eu/handle/10419/63430.

[bib0115] Sbardella A., Perruchas F., Napolitano L., Barbieri N., Consoli D. (2018). Green technology fitness. Entropy.

[bib0116] Sbardella A., Pugliese E., Pietronero L. (2017). Economic development and wage inequality: a complex system analysis. PLoS One.

[bib0117] Schetter, U. (2020). Quality Differentiation, Comparative Advantage, and International Specialization Across Products [paper]. Retrieved from https://papers.ssrn.com/sol3/papers.cfm?abstract_id=3091581.

[bib0118] Schetter, U. (2021) A structural ranking of economic complexity [paper]. Retrieved from https://www.econstor.eu/bitstream/10419/242451/1/vfs-2021-pid-50537.pdf.

[bib0119] Sciarra C., Chiarotti G., Ridolfi L., Laio F. (2020). Reconciling contrasting views on economic complexity. Nat. Commun..

[bib0120] Servedio, V.D., Buttà, P., Mazzilli, D., Tacchella, A., and Pietronero, L., (2018). A new and stable algorithm for economic complexity [paper]. Retrieved from https://pdfs.semanticscholar.org/f206/1e4f593a408acf6d63ea768414f392e17548.pdf. 10.3390/e20100783 PMC7512345 33265871

[bib0121] Smith A., Cannan E. (1776). Bantam Classic.

[bib0123] Stuart T.E. (1998). Network Positions and Propensities to Collaborate: an investigation of strategic alliance formation in a high-technology industry. Adm. Sci. Q..

[bib0124] Tacchella A., Cristelli M., Caldarelli G., Gabrielli A., Pietronero L. (2012). A new metrics for countries' fitness and products' complexity. Sci. Rep..

[bib0125] Tacchella A., Cristelli M., Caldarelli G., Gabrielli A., Pietronero L. (2013). Economic complexity: conceptual grounding of a new metrics for global competitiveness. *J. Econ. Dyn. Contro*l.

[bib0126] Teece D., Rumelt R., Dosi G., Winter S. (1994). Understanding corporate coherence - Theory and evidence. J. Econ. Behav. Organ..

[bib0127] Van Dam A., Frenken K. (2021). Variety, complexity and economic development. Res Policy.

[bib0128] Weitzman M.L. (1998). Recombinant growth. Q. J. Econ..

[bib0129] Zhu S., Yu C., He C. (2020). Export structures, income inequality and urban-rural divide in China. Appl. Geogr..

